# ELISA and Chemiluminescent Enzyme Immunoassay for Sensitive and Specific Determination of Lead (II) in Water, Food and Feed Samples

**DOI:** 10.3390/foods9030305

**Published:** 2020-03-08

**Authors:** Long Xu, Xiao-yi Suo, Qi Zhang, Xin-ping Li, Chen Chen, Xiao-ying Zhang

**Affiliations:** 1 College of Biological Science and Engineering, Shaanxi University of Technology, Hanzhong 723000, China; xu_lon@163.com (L.X.); 13259850610@163.com (Q.Z.); cchen2008@yahoo.com (C.C.); 2 Centre of Molecular and Environmental Biology, University of Minho, Department of Biology, Campus de Gualtar, 4710-057 Braga, Portugal; 3 College of Veterinary Medicine, Northwest A&F University, Yangling 712100, China; suoxiaoyi@nwafu.edu.cn (X.-y.S.); lxp67cqu@163.com (X.-p.L.)

**Keywords:** lead (II), ELISA, monoclonal antibody (mAb), isothiocyanobenzyl-EDTA (ITCBE), chemiluminescent enzyme immunoassay (CLEIA)

## Abstract

Lead is a heavy metal with increasing public health concerns on its accumulation in the food chain and environment. Immunoassays for the quantitative measurement of environmental heavy metals offer numerous advantages over other traditional methods. ELISA and chemiluminescent enzyme immunoassay (CLEIA), based on the mAb we generated, were developed for the detection of lead (II). In total, 50% inhibitory concentrations (IC_50_) of lead (II) were 9.4 ng/mL (ELISA) and 1.4 ng/mL (CLEIA); the limits of detection (LOD) were 0.7 ng/mL (ic-ELISA) and 0.1 ng/mL (ic-CLEIA), respectively. Cross-reactivities of the mAb toward other metal ions were less than 0.943%, indicating that the obtained mAb has high sensitivity and specificity. The recovery rates were 82.1%–108.3% (ic-ELISA) and 80.1%–98.8% (ic-CLEIA), respectively. The developed methods are feasible for the determination of trace lead (II) in various samples with high sensitivity, specificity, fastness, simplicity and accuracy.

## 1. Introduction

Environmental pollution from heavy metals is a worldwide issue. Lead has been widely used in the nuclear industry, glass manufacturing, battery industry, pipe industry, cosmetics industry, toy industry and paint industry [1]. Lead can be accumulated in the environment, as it cannot be rendered harmless through a chemical or bioremediation process. Plant leaves and roots are prone to accumulate toxic metals and can therefore be used for environmental monitoring, as a tool for assessing soil-contamination levels [2].

The major sources of lead exposure include piped drinking water, soldering from canned foods, beverages and traditional medicines. When indirectly ingested through contaminated food or inhalation, lead enters the food chain from the soil, water, deposition from the air, containers or dishes, and/or from food-processing equipment. Lead primarily accumulates in blood, soft tissues, bone and neurons, and this accumulation may cause behavioral changes, cognitive obstacles, blindness, encephalopathy, kidney failure and death. Children are more susceptible and vulnerable to lead due to its impact on the nervous system, as well as on development and behavioral performance [3]. Nowadays, lead pollution has become increasingly serious because of its excessive usage. The recent water contamination of lead in Flint, Michigan, remains a topical issue in public health. The decreased intelligence of children is directly positively correlated with blood lead and bone lead levels [4]. Although regulatory authorities have established safe levels of lead in foods (Table 1), the consensus is that there is no safe level of lead.

The spectroscopy methods for the detection of lead (II) included atomic absorption spectrometry (AAS) [5], atomic fluorescence spectrometry (AFS) [6] and multiple collectors inductively coupled plasma mass spectrometry (MC-ICP-MS) [7]. An AAS was used to detect Pb^2+^ in food with detection limit of 6 ng/mL [8]. These methods are sensitive and accurate, but costly and require intricate equipment and highly qualified technicians, making them unsuitable for onsite detection [9]. Recently, several sensors based on fluorophores, organic molecules and gold nanoparticles [10] have been reported to detect lead ions. A biomimetic sensor was applied in detecting Pb^2+^ in water, with a limit of detection of 9.9 ng/mL [11]. Lead (II) fluorescent sensors detections show high sensitivity, but require fluorophores and/or quenchers. Furthermore, the background signal could lead to serious interference due to its high fluorescence intensity [12]. Electrochemical sensors need tailor-made tactical materials and biological molecules, require skillful design and lengthy sample preparation and lack sufficient specificity. Therefore, the prospects of applying these sensors are limited [13,14].

Immunoassays have been applied for heavy-metal detection (e.g., cadmium, lead, chromium, uranium and mercury) [15], as they are quick, inexpensive, easy to perform, and highly sensitive and selective. ELISA and gold immunochromatographic assay (GICA) have been applied for detection of lead ions in water samples [16,17]. Chemiluminescent enzyme immunoassay (CLEIA), which has been widely used in pesticide and veterinary drug residue analysis, uses the energy generated by chemical reactions to excite luminescence, eliminating the need for external light sources. As a pilot attempt, this study aimed to develop CLEIA and the most commonly used ELISA for Pb^2+^ analysis in water, food and feed samples, to better address the current rapid and sensitive need on Pb^2+^ detection in environment and food contamination.

## 2. Materials and Methods 

### 2.1. Ethics Statement

All experimental animal protocols were reviewed and approved by the Ethics Committee of Shaanxi University of Technology for the Use of Laboratory Animals.

### 2.2. Chemicals and Reagents

Isothiocyanobenzyl-EDTA (ITCBE) was purchased from Dojindo (Kyushu, Japan). N, N’-dicyclohexylcarbodiimide (DCC), N-hydroxysuccinimide (NHS), dimethyl formamide (DMF), 3, 3’, 5, 5’-tetramethylbenzidine (TMB) and luminol were purchased from Solarbio (Beijing, China). HAT medium, keyhole hemocyanin (KLH) and bovine serum albumin (BSA) were purchased from Sigma (St. Louis, MO, USA). Goat anti-mouse IgG-HRP was purchased from Thermo (Waltham, MA, USA). Pb(NO_3_)_2_, HgSO_4_, 3CdSO_4_·8H_2_O, Cr_2_(SO_4_)_3_·6H_2_O, CuSO_4_, CoCl_2_·6(H_2_O), NiSO_4_.6H_2_O, ZnSO_4_·7H_2_O and FeSO_4_·7H_2_O were purchased from Sinopharma chemical reagent (Shanghai, China). OriginPro 8.1 (OriginLab, Northampton, MA, USA) was used for processing the analytical data.

### 2.3. Synthesis of Artificial Antigens of Lead

The ITCBE was conjugated to lead ions, BSA or KLH, using the DCC/NHS ester method. Briefly, equimolar amounts (0.06 mmol) of ITCBE, NHS and DCC were dissolved in 200 µL of DMF, and the same amount of lead nitrate was added to the mixture and stirred overnight. After centrifugation of the solution at 13,400× *g* for 10 min, the supernatant was added dropwise to 40 mg of BSA or KLH dissolved in 3 mL of 0.13 M NaHCO_3_ (pH 8.3), under stirring. After reaction for 4 h and centrifugation, the supernatant was dialyzed in phosphate buffered saline (PBS; 0.01 M; pH 7.4) for 4 days, with daily change of buffer.

UV spectra of lead (II)-ITCBE, BSA and lead (II)-ITCBE-BSA were tested at a wavelength ranging from 200 to 400 nm.

### 2.4. Production of Monoclonal Antibody

Four female BALB/C mice were immunized subcutaneously with 100 µg of lead (II)-ITCBE-KLH emulsified with an equal volume of Freund’s complete adjuvant. In the next two sequential booster immunizations, 50 µg of immunogen emulsified with the same volume of incomplete Freund’s adjuvant was given to each mouse, in the same way, at 2-week intervals. The fourth injection was administered intraperitoneally without adjuvant. Three days after the final booster injection, the mice were killed. Their spleen cells were removed and fused with mouse SP2/0 myeloma cells, using 50% PEG 4000 (*w*/*v*) as fusion agent. The mixture was spread in 96-well culture plates supplemented with hypoxanthine–aminopterin–thymidine (HAT) medium containing 20% fetal calf serum and peritoneal macrophages as feeder cells from BALB/C mice. The plates were incubated at 37 °C, with 5% CO_2_. After about 2 weeks, the supernatants were screened by an indirect competitive ELISA, using lead (II)-ITCBE-BSA as coating antigen. ITCBE, lead ions and lead (II)-ITCBE were tested as competitors. The hybridomas which were positive to lead (II)-ITCBE-BSA and negative to ITCBE-BSA were subcloned three times, using the limiting dilution method. Stable antibody-producing clones were expanded and cryopreserved in liquid nitrogen. Antibodies were collected and subjected to purification by ammonium sulfate precipitation. The purified mAb was stored at −20 °C, in the presence of 50% glycerol.

### 2.5. Indirect Competitive ELISA

The 96-well microtiter plates were coated with lead (II)-ITCBE-BSA conjugation (1 μg/mL, 100 µL/well) in carbonate buffer (CBS, 0.05 M, pH 9.6), and then incubated overnight at 4 °C. The plates were washed three times with PBST (PBS containing 0.05% Tween-20), using an automated plate washer, and blocked with blocking buffer (2% BSA in PBS, 200 µL/well) for 2 h, at 37 °C. After washing, diluted mAbs (stock concentration: 3.5 mg/mL, 1:32 000 dilution, 50 µL/well) were added to lead ions standard solutions (0.2, 1, 2, 5, 10, 20, 50, 100 and 200 ng/mL) or samples (50 µL/well) and incubated for 40 min, at 37 °C. After washing three times, the plates were incubated with goat anti-mouse IgG-HRP (stock concentration: 1.5 mg/mL, 1:8000, 100 µL/well), at 37 °C, for 40 min. Then, the washed plates were added with the substrate solution (TMB+H_2_O_2_, 100 µL/well). After 10 min of incubation, H_2_SO_4_ (2 M, 50 µL/well) was added, and the absorbance was measured at 450 nm. Normalized calibration curves were constructed in the form of (B/B_0_) × 100(%) vs. log C (lead ions) (where B and B_0_ were the absorbance of the analyte at the standard point and at zero concentration of the analyte, respectively.

### 2.6. Cross-Reactivity

The specificity of the mAb was investigated by cross-reactivity (CR). Different metal ions, including Hg^2+^, Cu^2+^, Ni^2+^, Zn^2+^, Cd^2+^, Fe^2+^, Co^2+^, Mg^2+^ and Ca^2+^ (in the form of their soluble chloride, nitrate, carbonate or sulfate salts), were analyzed. The standard solutions of cross-reacting chemicals were prepared in the concentration range of 0.001–1000 ng/mL. CR (%) = [IC_50_ for lead ions]/ [IC_50_ for competing chemical] × 100 (%).

### 2.7. Indirect Competitive CLEIA

The optimal concentrations of lead (II)-ITCBE-BSA and anti-lead antibody were selected, using ELISA, by checkerboard titration. The indirect competitive CLEIA was described as follows: 100 μL/well of lead (II)-ITCBE-BSA (1 μg/mL) in 0.05 M CBS (pH 9.6) was coated on the 96-well polystyrene microtiter plates and incubated at 4 °C overnight. The following day, the plate was washed three times, using PBST, and blocked with 2% BSA in PBS (200 μL per well), at 37 °C, for 2 h. After a further washing step, 50 μL of diluted mAb (stock concentration: 3.5 mg/mL, 1:32 000 dilution) and 50 μL of lead ions standard solution were added to each well and incubated at 37 °C, for 40 min. Lead ions standard solution was prepared by diluting with PBS at a series of concentrations (0.2, 0.5, 1, 2, 5, 10, 20, 50, 100 and 200 ng/mL). After washing with PBST, the plates were incubated, and goat anti-mouse IgG-HRP (stock concentration: 1.5 mg/mL, 1:8000, 100 μL per well) was added and incubated at 37 °C, for 40 min. Finally, 100 µL of substrate solution prepared freshly was added into each well and incubated for 5 min, in the dark. Then chemiluminescence intensity was monitored on Synergy H1. The standard curve was evaluated by plotting chemiluminescence intensity against the logarithm of each concentration and fit to a logistic equation, using OriginLab 8.1 program.

### 2.8. Graphite Furnace Atomic Absorption Spectrometry (GFAAS)

The operating parameters of the GFAAS system were as follows: lead hollow lamp current 30 mA, wavelength 283.3 nm, shielding gas (Ar) flow rate 1500 mL/min, carrier gas (Ar) flow rate 500 mL/min, and ashing temperature and time were 450 °C and 9 s. The atomization temperature, heating rate and heating time were 2250 °C, 2200 °C/s and 3 s, respectively. The carrier solution was HNO_3_ (5.0%, *v/v*). The calibration curve for lead ions was constructed with standards of 0, 0.1, 0.2, 0.4, 0.6, 0.8, 1.0, 1.4, 1.8, 2.4 and 3.0 μg/L.

### 2.9. Sample Preparation and Spiked Experiment

Spiked samples were used to examine the assay accuracy and precision.

Water samples, including ultrapure water, tap water and river water, were collected from different sites in Yangling, Shaanxi province, China. Water samples (100 mL) were added with Pb standard solution (1 mg/mL) at the final concentration of 100, 200 and 500 ng/mL. Ultrapure water and tap water were analyzed without any dilution and sample preparation. The river water was filtrated with a 0.45 µm nylon membrane filter and adjusted to pH 7.0 before analysis.

Milk samples were collected from the local market. Milk samples (100 mL) were added with Pb standard solution (1 mg/mL) at the final concentration of 100, 200 and 500 ng/mL. Then the samples were boiled to remove the denatured protein and fat, and then an equal volume of acetate buffer solution (0.1 M, pH 5.7) was added for precipitation. After being maintained at room temperature for 2 h, the mixture was centrifuged at 13,400× *g* for 10 min. The pH of the supernatants was adjusted to 7.0 with 1 M NaOH and diluted with pure water for analysis.

Chicken, rice and feed samples (1.0 g) were homogenized and added with Pb standard solution (1 mg/mL) at the final amounts of 100, 200 and 500 ng. Then the samples were extracted by acid leach method. The samples were soaked with 20% HNO_3_, overnight, at room temperature, followed by boiling until fully dissolved. After cooling, the solution was centrifuged, and the supernatant was adjusted to a pH value of 7.0 with 1 M NaOH and diluted with pure water for further analysis.

### 2.10. Pretreatment of Samples for GFAAS

Water samples (10 mL) were added with Pb standard solution (1 mg/mL) at the final amounts of 1, 2 and 5 μg. Then the samples were mixed with 50% HCl (1 mL), 0.8 mL of a solution containing KBrO_3_ (0.1 M) and KBr (0.084 M). After reaction for 15 min, an appropriate amount of hydroxylamine hydrochloride/sodium chloride (both at a concentration of 120 g/L) solution was added until the yellow color disappeared. The solution was further diluted with pure water, to 200 mL, and determined by GFAAS.

Chicken, rice and feed samples were pretreated, using a microwave-assisted acid-digestion procedure. Samples (1.0 g) were homogenized and added with Pb standard solution (1 mg/mL) at the final amounts of 100, 200 and 500 ng. Then the samples were transferred into polytetrafluoroethylene (PTFE) flasks, and then HNO_3_ (8 mL) and H_2_O_2_ (2 mL) were added to each flask and kept for 15 min, at room temperature. The flasks were sealed and subjected to microwave digestion. Finally, the samples were diluted with pure water, to 200 mL, for GFAAS detection.

## 3. Results

### 3.1. Characterization of the Artificial Antigen and the Monoclonal Antibody

**Figure 1:**
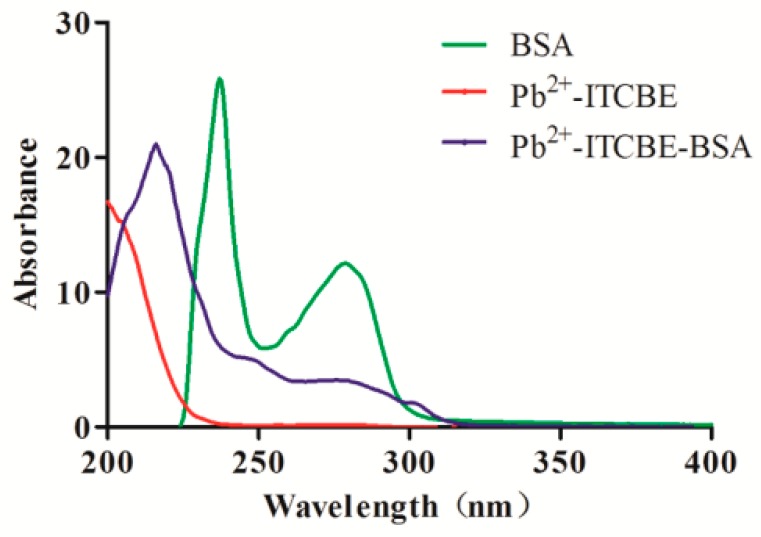
UV absorbance spectra of lead (II)-ITCBE, BSA and lead (II)-ITCBE-BSA.

Lead (II)-ITCBE, BSA and Lead (II)-ITCBE-BSA spectra were recorded from 200 to 400 nm. BSA exhibits a characteristic ultraviolet absorption peak at 229 and 278 nm, and lead (II)-ITCBE-BSA exhibits a characteristic ultraviolet absorption peak at 215 nm. The shift of the ultraviolet absorption peak proved that the artificial antigen synthesis was successful (see Figure 1).

The anti-lead mAb was purified from mice ascites, using ammonium sulfate precipitation and protein G column affinity chromatography with an obtained concentration of 3.5 mg/mL. The isotype of mAb was IgG1 with a kappa light chain.

### 3.2. Development of ic-ELISA

**Figure 2:**
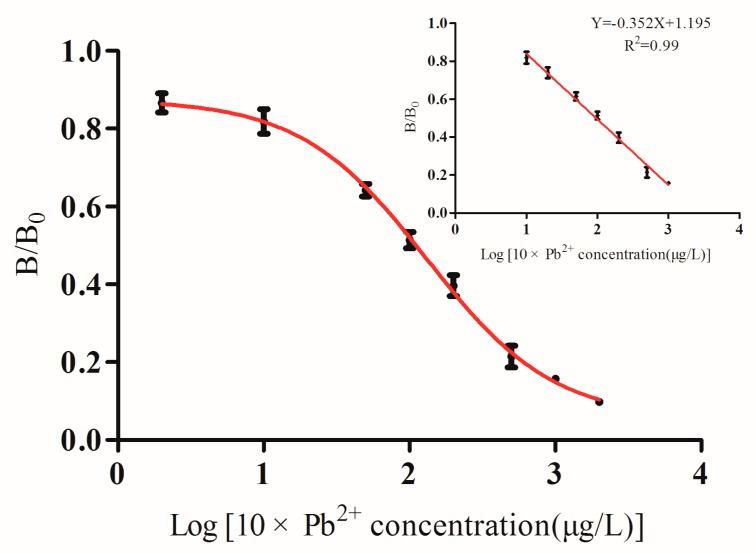
Standard curve of the competitive ELISA for lead ions.

Sensitivity of ELISA was determined under optimal conditions. In the representative competitive inhibition curve for lead ions (see Figure 2), the regression curve equation of the anti-lead mAb was Y = −0.352X + 1.195 (R^2^ = 0.990, *n* = 3), with an IC_50_ value of 9.4 ng/mL and limit of detection (IC_10_ value) of 0.7 ng/mL. The ELISA could be used for Pb^2+^ detection with a linear range from 1 to 100 ng/mL.

### 3.3. Cross-Reactivity

The obtained mAb did not recognize the other eight common metal ions (see Table 2).

### 3.4. Chemiluminescence Immunoassay

**Figure 3:**
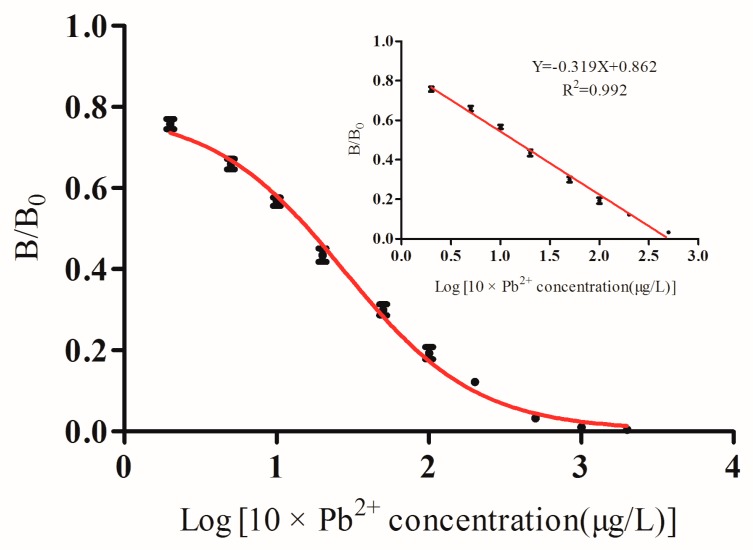
Standard curve of the competitive CLEIA for lead ions.

The sensitivity of ic-CLEIA was determined under optimal conditions. The representative competitive inhibition curve (see Figure 3) revealed the regression curve equation of Y = −0.319X + 0.862 (R^2^ = 0.992, *n* = 3), with IC_50_ value of 1.4 ng/mL, the limit of detection (IC_10_ value) of 0.1 ng/mL and the linear range from 0.2 to 50 ng/mL.

### 3.5. GFAAS Analysis of Pb^2+^

**Figure 4:**
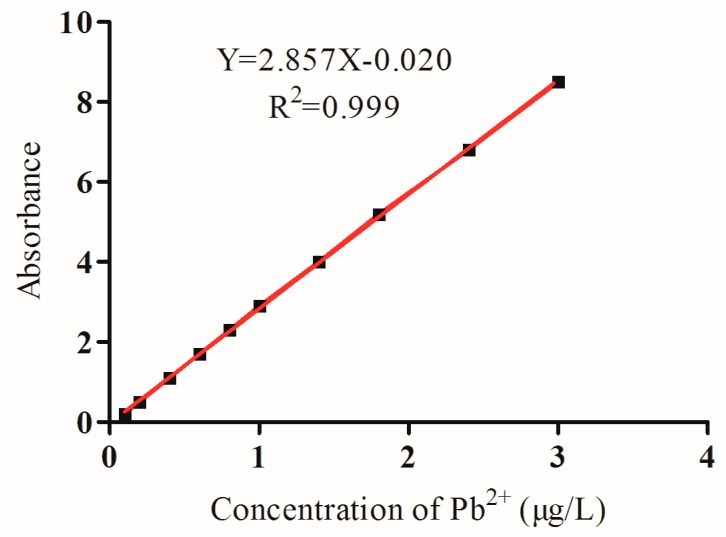
Standard curve of the GFAAS for lead ions.

The sensitivity of GFAAS was determined under optimal conditions. The regression curve equation was Y = 2.857X − 0.020 (*R^2^* = 0.999, *n* = 3; see Figure 4). The linearity ranged from 0 to 3.0 μg/L. The limit of quantification was 0.86 μg/L.

### 3.6. Precision and Recovery in Sample Test

The spiked chicken, rice, chicken feed, rat feed, milk and tap water samples containing different concentrations of lead ions (100, 200 and 500 ng/g, respectively) were detected by using the proposed ic-ELISA and ic-CLEIA, respectively, and both methods showed high recoveries and low coefficients of variation (see Table 3). The recovery of the spiked samples suggested that the CLEIA is suitable as a rapid and reliable method to detect lead ions in several matrices.

### 3.7. Comparison of ELISA, CLEIA and GFAAS Results for Lead (II) in Samples 

**Figure 5:**
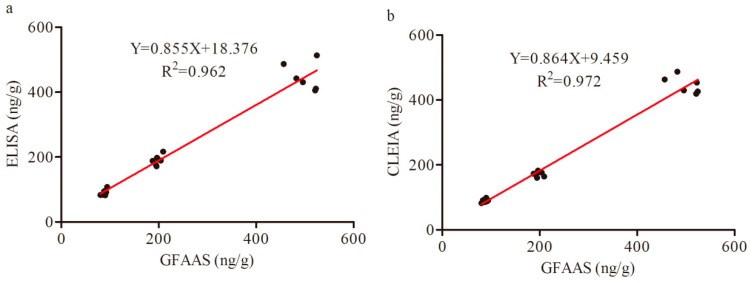
Correlation of ELISA and CLEIA to GFAAS on lead ions analysis.

The linear regression curves of ELISA (see Figure 5a) and CLEIA (see Figure 5b) showed good correlation coefficients square of 0.962 and 0.972, respectively, as compared to GFAAS, indicating that the two methods developed could achieve reliable and accurate determination of lead (II) ions in samples.

## 4. Discussion

The small size and simple structure of heavy metal ions result in poor immunogenicity; as such, they are classified as incomplete antigen. To generate complete antigens for immunological assays, a highly effective bifunctional chelating agent (ITCBE) was selected to connect the lead ion and the carrier protein, which has a large relative molecular mass, reduced toxicity and enhanced immunogenicity [22,23]. The mAb we obtained is superior in sensitivity and specificity as compared to the mAb generated by using the conjugation of lead and S-2-(4-aminobenzyl) diethylenetriamine penta-acetic acid (DTPA) as immunogen, which was applied in ELISA with a limit of detection of 11.6 ng/mL and cross-activity less than 3% [24].

Sample pretreatment is a primary factor for enriching heavy metals and minimizing matrix interference in practical application, as in real detection conditions, lead ions often bind tightly to larger molecules, such as proteins, carbohydrates and colloids [17]. Several methods have been used for heavy-metal sample pretreatment. Microwave digestion method was often used to extract heavy metals from solid samples, including chicken, fish, feces and soil, with high accuracy and recovery rate, but it has limitations in real-time and high throughput detection [25]. A recent study on extracting lead ions in skin-whitening cosmetics, using microwave digestion coupled with plasma atomic emission spectrometry, showed a detection limit of 3.8 μg/kg [26]. The dry ash method is usually used to enrich heavy metals from food and plant samples; however, it demonstrated low accuracy, low recovery rate and high blank value, and it is not suitable for food containing highly volatile inorganic salt [27]. Dry ash extraction has been used for GFAAS-based lead measurement from green vegetables with obtained recovery ranging from 67% to 103% [28]. To better separate the lead ions, we used the acid leach method to enriched lead ions in samples, and have achieved high recovery and a good variable coefficient (Table 3). Furthermore, the acid leach method is easy to operate and has no loss of element, as compared to the other methods, such as microwave digestion, wet digestion and dry ash.

## 5. Conclusions

In this study, a monoclonal antibody against lead (II) was raised by immunizing Balb/c mouse and hybridoma technique. The LOD of ic-ELISA and ic-CLEIA were 0.7 and 0.1 ng/mL, respectively. The ic-ELISA and ic-CLEIA demonstrated low coefficient of variation. Compared to GFAAS, the two developed methods showed a wide detection range, and the ic-CLEIA showed even more sensitivity compared to the ic-ELISA.

Collectively, ic-ELISA and ic-CLEIA were developed for handy, sensitive and specific detection of lead (II) ions in water, food and feed.

## Figures and Tables

**Table 1:** Permitted maximum amount of lead.

	Fresh Vegetable (mg/kg)	Cereals (mg/kg)	Fresh Fruits (mg/kg)	Mushroom (mg/kg)	Beans (mg/kg)	Livestock and Poultry Meat (mg/kg)	Livestock and Poultry Gut (mg/kg)	Fish (mg/kg)	Salt (mg/kg)	Drinking Water (mg/L)
CAC [18]	0.1	0.2	0.1	-	0.2	0.1	0.5	0.3	2	0.01
EFSA [19]	0.1	0.2	0.1	0.3	0.2	0.1	0.5	0.3	-	0.01
CFDA [20]	0.1	0.2	0.1	1	0.2	0.2	0.5	0.5	2	0.01
FSANZ [21]	0.1	0.2	0.1	-	0.2	0.1	0.5	0.5	-	0.05

Notes: CAC: Codex Alimentarius Commission; CFDA; Chinese Food and Drug Administration; EFSA: European Food Safety Authority; FSANZ: Food Standards Australia New Zealand.

**Table 2:** Cross-reactivity of anti-lead IgG with other metal ions (*n* = 3).

Compounds	IC_50_ (µg/L)	Cross-Reactivity (%)
Pb^2+^ -ITCBE	9.4	100
Hg^2+^ -ITCBE	>1×10^3^	<0.943
Cd^2+^ -ITCBE	>1×10^3^	<0.943
Cr^3+^ -ITCBE	>1×10^3^	<0.943
Cu^2+^ -ITCBE	>1×10^3^	<0.943
Co^2+^ -ITCBE	>1×10^3^	<0.943
Ni^2+^ -ITCBE	>1×10^3^	<0.943
Zn^2+^ -ITCBE	>1×10^3^	<0.943
Fe^2+^ -ITCBE	>1×10^3^	<0.943

**Table 3:** Recovery ratio of Pb^2+^ from different samples (*n* = 4).

Samples		ELISA			CLEIA			AAS		
	Spiked	Mea-sured	Recovery	RSD	Mea-sured	Recovery	RSD	Mea-sured	Recovery	RSD
	(ng/g)	(ng/g)	(%)	(%)	(ng/g)	(%)	(%)	(ng/g)	(%)	(%)
Chicken	0	<LOD	--	---	<LOD	--	---	<LOD	--	---
	100	91.0	91.0	10.1	88.2	88.2	16.0	92.1	92.1	8.9
	200	197.5	98.7	11.4	182.2	91.1	2.5	196.4	98.2	0.2
	500	442.3	88.5	15.0	487.4	97.5	4.3	482.5	96.5	0.3
Rice	0	<LOD	--	---	<LOD	--	---	<LOD	--	---
	100	82.1	82.1	5.5	98.8	98.8	12.1	90.2	90.2	9.1
	200	189.1	94.6	8.4	176.4	88.2	9.3	204.0	102.0	4.7
	500	405.2	81.0	1.7	419.3	83.9	11.7	521.2	104.2	0.2
Chicken feed	0	<LOD	--	---	<LOD	--	---	<LOD	--	---
	100	83.8	83.8	6.8	91.3	91.3	6.8	83.3	83.3	8.7
	200	216.5	108.3	12.1	164.5	82.2	12.0	209.2	104.6	0.3
	500	430.8	86.1	6.8	429.9	85.9	10.6	495.7	99.1	0.2
Rat feed	0	<LOD	--	---	<LOD	--	---	<LOD	--	---
	100	83.0	83.0	9.6	82.0	82.0	11.0	80.3	80.3	1.4
	200	171.9	85.9	8.5	175.2	87.6	8.2	195.5	97.7	0.3
	500	410.5	82.1	9.6	453.5	90.7	10.9	522.5	104.5	1.6
Milk	0	<LOD	--	---	<LOD	--	---	<LOD	--	---
	100	94.5	94.5	12.0	85.6	85.6	12.3	88.01	88.01	8.0
	200	175.0	87.5	10.5	160.3	80.1	2.3	194.6	97.3	0.6
	500	486.7	97.3	14.1	463.2	92.6	8.4	456.5	91.3	0.3
Tap water	0	<LOD	--	---	<LOD	--	---	<LOD	--	---
	100	107.7	107.7	10.2	90.9	90.9	8.7	94.2	94.2	9.3
	200	188.3	94.1	9.8	172.8	86.4	5.0	187.4	93.7	0.5
	500	513.0	102.6	3.6	426.3	85.3	4.2	524.5	104.9	0.2

## References

[1] Molera J., Pradell T., Salvadó N., Vendrell-Saz M. (2001). Interactions between Clay Bodies and Lead Glazes. J. Am. Ceram. Soc..

[2] Szczyglowska M., Bodnar M., Namiesnik J., Konieczka P. (2014). The Use of Vegetables in the Biomonitoring of Cadmium and Lead Pollution in the Environment. Crit. Rev. Anal. Chem..

[3] Sang Yong E., Young-Sub L., Seul-Gi L., Mi-Na S., Byung-Sun C., Yong-Dae K., Ji-Ae L., Myung-Sil H., Ho-Jang K., Yu-Mi K. (2018). Lead, Mercury, and Cadmium Exposure in the Korean General Population. J Korean Med. Sci..

[4] Wasserman G.A., Factor-Litvak P., Liu X., Todd A.C., Graziano J.H. (2003). The Relationship Between Blood Lead, Bone Lead and Child Intelligence. Child Neuropsychol..

[5] Oliveira de T.M., Peres J.A., Felsner M.L., Justi K.C. (2017). Direct determination of Pb in raw milk by graphite furnace atomic absorption spectrometry (GF AAS) with electrothermal atomization sampling from slurries. Food Chem..

[6] da Silva D.L.F., da Costa M.A.P., Silva L.O.B., dos Santos W.N.L. (2019). Simultaneous determination of mercury and selenium in fish by CVG AFS. Food Chem..

[7] Chen K.y., Fan C., Yuan H.l., Bao Z.a., Zong C.l., Dai M.n., Ling X., Yang Y. (2013). High-Precision In Situ Analysis of the Lead Isotopic Composition in Copper Using Femtosecond Laser Ablation MC-ICP-MS and the Application in Ancient Coins. Spectrosc. Spect. Anal..

[8] Da Col J.A., Domene S.M.A., Pereira-Filho E.R. (2009). Fast Determination of Cd, Fe, Pb, and Zn in Food using AAS. Food Anal. Methods.

[9] Wei C., Shunbi X., Jin Z., Dianyong T., Ying T. (2018). Immobilized-free miniaturized electrochemical sensing system for Pb^2+^ detection based on dual Pb^2+^ -DNAzyme assistant feedback amplification strategy. Biosens. Bioelectron..

[10] Wang X.Y., Niu C.G., Guo L.J., Hu L.Y., Wu S.Q., Zeng G.M., Li F. (2017). A Fluorescence Sensor for Lead (II) Ions Determination Based on Label-Free Gold Nanoparticles (GNPs)-DNAzyme Using Time-Gated Mode in Aqueous Solution. J. Fluoresc..

[11] Chu W., Zhang Y., Li D., Barrow C.J., Wang H., Yang W. (2015). A biomimetic sensor for the detection of lead in water. Biosens. Bioelectron..

[12] Liu C.W., Huang C.C., Chang H.T. (2009). Highly Selective DNA-Based Sensor for Lead(II) and Mercury(II) Ions. Anal. Chem..

[13] Tang S.r., Lu W., Gu F., Tong P., Yan Z., Zhang L. (2014). A novel electrochemical sensor for lead ion based on cascade DNA and quantum dots amplification. Electrochim. Acta.

[14] Zhang H., Jiang B., Xiang Y., Su J., Chai Y., Yuan R. (2011). DNAzyme-based highly sensitive electronic detection of lead via quantum dot-assembled amplification labels. Biosens. Bioelectron..

[15] Xiang J.J., Zhai Y.f., Tang Y., Wang H., Liu B., guo C.W. (2010). A competitive indirect enzyme-linked immunoassay for lead ion measurement using mAbs against the lead-DTPA complex. Environ. Pollut..

[16] Tang Y., Zhai Y.F., Xiang J.J., Wang H., Guo C.W. (2010). Colloidal gold probe-based immunochromatographic assay for the rapid detection of lead ions in water samples. Biosens. Bioelectron..

[17] Mandappa I.M., Ranjini A., Haware D.J., Manonmani H.K. (2014). Immunoassay for lead ions: analysis of spiked food samples. J. Immunoassay.

[18] Codex Alimentarius Commission (CSC) (1995). Codex general standard for contaminants and toxins in food and feed. Codex stan.

[19] The commission of the European Community (2006). Commission regulation (EC) NO 1881/2006 setting maximum levels for certain contaminants in foodstuffs. OJEC.

[20] National food safety standard for maximum levels of contaminants in foods. http://www.nhc.gov.cn/ewebeditor/uploadfile/2013/01/20130128114248937.pdf..

[21] Australia New Zealand Standard 1.4.1 contaminants and natural toxicants. https://www.foodstandards.gov.au/code/Documents/Sched%2019%20Contaminant%20MLs%20v157.pdf.

[22] Perrin C.L., Kim Y.J. (2000). Symmetry of Metal Chelates. Inorg. Chem..

[23] Love R.A., Villafranca J.E., Aust R.M., Nakamura K.K., Jue R.A., Major J.G., Radhakrishnan R., Butler W.F. (1993). How the anti-(metal chelate) antibody CHA255 is specific for the metal ion of its antigen: X-ray structures for two Fab’/hapten complexes with different metals in the chelate. Biochemistry.

[24] Zhu X., Hu B., Lou Y., Xu L., Yang F., Yu H., Blake D.A., Liu F. (2007). Characterization of monoclonal antibodies for lead chelate complexes: applications in antibody-based assays. J. Agric. Food Chem..

[25] Safari Y., Karimaei M., Sharafi K., Arfaeinia H., Moradi M., Fattahi N. (2017). Persistent sample circulation microextraction combined with graphite furnace atomic absorption spectroscopy for trace determination of heavy metals in fish species marketed in Kermanshah, Iran and human health risk assessment. J. Sci. Food Agric..

[26] Alqadami A.A., Mu N., Abdalla M.A., Khan M.R., Alothman Z.A., Wabaidur S.M., Ghfar A.A. (2017). Determination of heavy metals in skin-whitening cosmetics using microwave digestion and inductively coupled plasma atomic emission spectrometry. IET Nanobiotechnol..

[27] Hadiani M.R., Farhangi R., Soleimani H., Rastegar H., Cheraghali A.M. (2014). Evaluation of heavy metals contamination in Iranian foodstuffs: Canned tomato paste and tomato sauce (ketchup). Food Addit. Contam. Part B.

[28] Baxter M.J., Burrell J.A., Crews H.M., Massey R.C., McWeeny D.J. (1989). A procedure for the determination of lead in green vegetables at concentrations down to 1 μg/kg. Food Addit. Contam..

